# The Role of Soil Microorganisms in Plant Mineral Nutrition—Current Knowledge and Future Directions

**DOI:** 10.3389/fpls.2017.01617

**Published:** 2017-09-19

**Authors:** Richard Jacoby, Manuela Peukert, Antonella Succurro, Anna Koprivova, Stanislav Kopriva

**Affiliations:** Botanical Institute, Cluster of Excellence on Plant Sciences (CEPLAS), University of Cologne Cologne, Germany

**Keywords:** plant–microbe interactions, plant nutrition, microbiome, root exudates, natural variation, mathematical modeling

## Abstract

In their natural environment, plants are part of a rich ecosystem including numerous and diverse microorganisms in the soil. It has been long recognized that some of these microbes, such as mycorrhizal fungi or nitrogen fixing symbiotic bacteria, play important roles in plant performance by improving mineral nutrition. However, the full range of microbes associated with plants and their potential to replace synthetic agricultural inputs has only recently started to be uncovered. In the last few years, a great progress has been made in the knowledge on composition of rhizospheric microbiomes and their dynamics. There is clear evidence that plants shape microbiome structures, most probably by root exudates, and also that bacteria have developed various adaptations to thrive in the rhizospheric niche. The mechanisms of these interactions and the processes driving the alterations in microbiomes are, however, largely unknown. In this review, we focus on the interaction of plants and root associated bacteria enhancing plant mineral nutrition, summarizing the current knowledge in several research fields that can converge to improve our understanding of the molecular mechanisms underpinning this phenomenon.

## Introduction

### The Interconnection of Plants with Soil Microbes

Although plant physiologists sometimes view soil as simply a source of nutrients to plants, it is actually a complex ecosystem hosting bacteria, fungi, protists, and animals (Bonkowski et al., 2009; Muller et al., 2016). Plants exhibit a diverse array of interactions with these soil-dwelling organisms, which span the full range of ecological possibilities (competitive, exploitative, neutral, commensal, mutualistic). Throughout modern plant science, most interaction studies have focused on alleviating pathogenic effects such as herbivory and infection (Strange and Scott, 2005; Zhang et al., 2013), or attenuating abiotic stress conditions (Yaish et al., 2016; Meena et al., 2017). However, there has also been longstanding interest in characterizing the positive ecological interactions that promote plant growth. For instance, mycorrhizal fungi as well as the bacteria present in nodulated legumes were both recognized as root symbionts from the second half of 19th century (Morton, 1981). Already in the 1950s, crop seeds were coated with bacterial cultures (*Azotobacter chroococcum* or *Bacillus megaterium*) to improve growth and yield (Brown, 1974). By the 1980s many different bacterial strains, mainly *Pseudomonas* but also *Azospirillum*, had been described as having plant growth promoting effects (Burr et al., 1978; Teintze et al., 1981; Lin et al., 1983). Since the 2000s, research focus has somewhat shifted away from individual microbial strains, and toward documenting the abundance and diversity of the root microbiome through metagenomics. Results from such sequencing studies have shown that the rhizospheric niche is a hotspot of ecological richness, with plant roots hosting an enormous array of microbial taxa (Bulgarelli et al., 2013). In the last few years, research has swung toward assembling rationally designed synthetic communities (SynComs) that comprise strains representing the dominant rhizospheric taxa, with the aim of re-capitulating favorable microbial functions under controlled experimental conditions (Busby et al., 2017). A major goal of this research field is to gain a mechanistic understanding of how soil microbes boost plant growth and defense, and then to use this knowledge to inform the optimal design of microbial communities tailored to carry out specific functions.

### Microbial Traits and the Bioavailability of Nutrients for Plants

Three mechanisms are usually put forward to explain how microbial activity can boost plant growth: (1) manipulating the hormonal signaling of plants (Verbon and Liberman, 2016); (2) repelling or outcompeting pathogenic microbial strains (Mendes et al., 2013); and (3) increasing the bioavailability of soil-borne nutrients (van der Heijden et al., 2008). This review will focus on the third mechanism, whereby soil microbes metabolize recalcitrant forms of soil-borne nutrients to liberate these elements for plant nutrition. In natural ecosystems, most nutrients such as N, P, and S are bound in organic molecules and are therefore minimally bioavailable for plants. To access these nutrients, plants are dependent on the growth of soil microbes such as bacteria and fungi, which possess the metabolic machinery to depolymerize and mineralize organic forms of N, P, and S. The contents of these microbial cells are subsequently released, either through turnover and cell lysis, or via protozoic predation (Bonkowski, 2004; Richardson et al., 2009). This liberates inorganic N, P, and S forms into the soil, including ionic species such as ammonium, nitrate, phosphate, and sulfate that are the preferred nutrient forms for plants (van der Heijden et al., 2008). In natural settings, these microbial nutrient transformations are key drivers of plant growth, and can sometimes be the rate-limiting step in ecosystem productivity (Schimel and Bennett, 2004).

### Fertilization Practices and Environmental Sustainability

**FIGURE 1:**
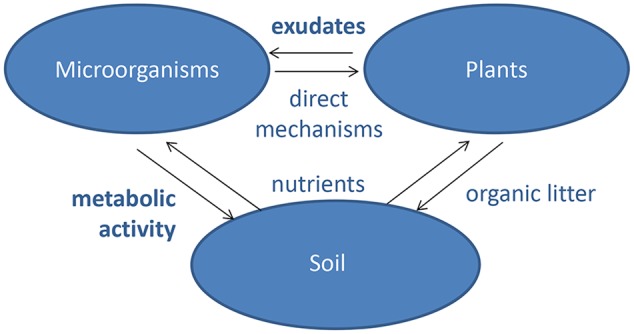
Interactions between plants, microbiota, and soil. Both plants and microorganisms obtain their nutrients from soil and change soil properties by organic litter deposition and metabolic activities, respectively. Microorganisms have a range of direct effects on plants through, e.g., manipulation of hormone signaling and protection against pathogens. Plants communicate with the microorganisms through metabolites exuded by the roots. The major knowledge gaps for understanding the mechanisms of plant–microbe interactions in the rhizosphere are shown in bold.

In most contemporary agricultural systems, macronutrients are provided through the application of mineral fertilizers. However, unsustainable fertilization practices are contributing to the large-scale alterations of Earth’s biogeochemical cycles, through mechanisms such as soil degradation, waterway eutrophication, and greenhouse gas emissions (Amundson et al., 2015; Steffen et al., 2015). Furthermore, known reserves of phosphate rock are rapidly diminishing and predicted to be exhausted within a few decades (Cordell and White, 2014), while N-fertilizer production via the energy-intensive Haber–Bosch process relies upon fossil fuels and thus exacerbates global warming and natural resource depletion (Erisman et al., 2013). Due to the scale and severity of these fertilizer-induced problems, a current research priority is for agricultural science to develop alternative methods of sustaining plant nutrition with dramatically lower inputs of mineral fertilizers (Foley et al., 2011). One such possibility is to replace mineral fertilizers by organic inputs, and to supplement plants with specific root-associated microbes that depolymerize and mineralize the organic-bound nutrients. The logic of this idea is that organic inputs can be obtained more sustainably than mineral fertilizers, because myriad agricultural, industrial and municipal processes produce huge volumes of nutrient-rich “waste” that are currently disposed of, but could potentially be composted and applied as fertilizers (Paungfoo-Lonhienne et al., 2012). Another factor is that organically bound nutrients are more stable in the soil compared to mineral fertilizers, and therefore less prone to leaching and volatilization (Reganold and Wachter, 2016). Bio-fertilizers are already used in organic farming systems, but there is currently little mechanistic insight behind the choice of plant cultivars and microbial inoculants (Bender et al., 2016; Reganold and Wachter, 2016). This lack of precision occurs because of two major knowledge gaps: (1) it is unclear what strategies plants use to recruit beneficial microbes, and how much genetic variation exists for this trait; and (2) There is insufficient knowledge of which particular microbes are best partners for boosting plant nutrition from organic sources of N, P, and S (**Figure 1**).

We aim to understand how microbes contribute to plant nutrition, and also how plants shape their microbiome to maximize the nutritional benefits of this interaction. In this review, we summarize current progress in approaches toward dissection of the interconnection of plants and bacteria in mineral nutrition, with emphasis on metabolic capacities of plants and microbes. When reviewing this field, it must be mentioned that there is a significant body of literature studying how certain plants can receive nutritional benefits via symbiotic associations with mycorrhiza and nodulating bacteria. To avoid overlaps with some excellent recent reviews (Smith and Smith, 2011; Udvardi and Poole, 2013; Garcia et al., 2016; Kamel et al., 2017), we do not focus on these well-characterized symbiotic interactions. Instead, we focus on how plant nutrition can be linked to the entire rhizospheric microbiome, an emerging field that is currently undergoing rapid growth. We focus the review on bacteria, although most of the concepts are valid also for other soil organisms, particularly fungi. We are making a case for a multidisciplinary approach that combines plant and microbial genetics with biochemistry and metabolic modeling. Together, these tools can enhance our mechanistic understanding of the interactions between plants and microbes, and how these processes can be optimized to drive plant nutrition with lower applications of mineral fertilizers.

## Effects of Plant Natural Variation on the Rhizosphere Microbiome

To selectively breed plants for optimized nutritional interactions with soil microbes, the genetic components of this trait must first be discovered. Sequence analyses showed differences between the composition of bacterial taxa in soil and plant rhizosphere or the endophytic fraction, showing that plants select for specific bacterial taxa and thus exert some control over their microbiomes (Bulgarelli et al., 2012; Turner et al., 2013; Zgadzaj et al., 2016). The next question is then to define the key genetic determinants that underpin how different plant genotypes interact with rhizospheric bacteria. Decades of research have shown that the susceptibility to pathogenic microorganisms is highly dependent on plant genome, between different species as well as within accessions of the same one (Zhang et al., 2013). Similarly, Arabidopsis accessions showed large variation in supporting growth of rhizospheric bacterium *Pseudomonas fluorescens* in a hydroponic system (Haney et al., 2015). Indeed, sequence analyses have confirmed different microbiome structures across plant taxa, with bigger differences in more distant species and with also a larger contribution of environment and soil to the variation (Turner et al., 2013; Schlaeppi et al., 2014; Zgadzaj et al., 2016). When comparing accessions or varieties of the same species, genotypic effects on microbiome structure have been seen amongst Arabidopsis, maize, and barley (Bulgarelli et al., 2012, 2015; Peiffer et al., 2013). Regarding leaf microbiota, clear differences were shown for leaf microbiomes across 196 Arabidopsis accessions (Horton et al., 2014). The variation driven by plant-genome was particularly high for the most abundant operational taxonomic units (OTU). The variation was further explored by genome-wide association study (GWAS) using the number of reads for individual OTUs as quantitative phenotypes (Horton et al., 2014). There, GWAS revealed that many of the significant SNPs linked to bacterial OTU structure were categorized as defense response, which was the most overrepresented gene ontology term among the candidate genes. In addition, genes involved in cell wall synthesis and kinase activity were enriched (Horton et al., 2014). Although several candidate genes affecting the leaf microbiome were identified, further confirmatory tests of mutants of these genes have not been reported and therefore the functionality of the genes in shaping the microbiome remains to be demonstrated. The leaf microbiome GWAS can be of major importance for understanding the processes in rhizosphere, because the leaf and root microbiomes are overlapping (Bai et al., 2015) and might be shaped by similar processes. In an alternative approach to GWAS, Bodenhausen et al. (2014) monitored changes to SynComs inoculated onto leaves of Arabidopsis accessions and mutants of *a priori* selected genes (Bodenhausen et al., 2014). Again, a clear genotype effect upon microbial taxonomic composition has been observed in the 10 accessions and in several mutants. In three mutants, the effects were consistent and reproducible, two mutants were involved in cuticle synthesis and one in ethylene signaling (*ein2*) (Bodenhausen et al., 2014). Given that only some 40 mutants and highly simplified SynComs were tested, the approach seems to be promising for analysis of root microbiome as well, particularly if mutants in nutrient uptake and assimilation would be investigated.

### GWAS of Bacteria-Mediated Plant Traits

**FIGURE 2:**
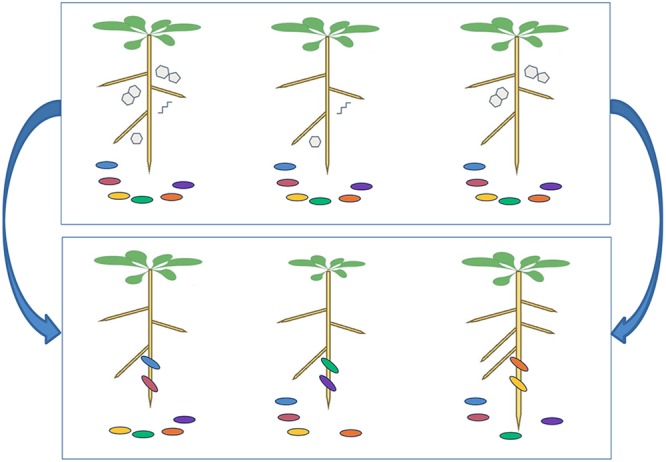
Plant natural variation in root exudates leading to distinctive microbial communities. We postulate that plant genotype has a strong effect on microbial community composition, mediated via root exudate composition. Consider three plant genotypes with differing root exudate profiles. At time 0 (upper panel), the three plants are transplanted from a sterile system onto a common soil where they are proximate to the same set of microbial strains. At time *t* (lower panel), the three plant genotypes have recruited distinctive microbial strains to their roots, which confer differential growth-promoting effects manifesting in different plant sizes.

The sequence analyses, however, explore only the taxonomical composition of plant microbiome without taking into account the whole bacterial genomes or addressing the functions these microbes are performing. The best attempt so far to assess how plant genotype affects functional interaction with rhizobacteria is the analysis of variation in susceptibility of Arabidopsis accessions to the plant growth-promoting rhizobacterium *Pseudomonas simiae* WCS417r (Wintermans et al., 2016). The authors cultivated 302 accessions with and without the bacterium, which promotes changes in root architecture and growth through volatile emission. The accessions showed large difference in all three phenotypes scored: fresh weight gain, proliferation of lateral roots, and elongation of the primary root (Wintermans et al., 2016). Statistical GWAS analysis resulted in several highly significant associations, but despite some good correlation between the fresh weight and root architecture data, none of the positive SNPs were found with multiple phenotypes. The analysis led to identification of several candidate genes, but without further verification or confirmatory experiments (Wintermans et al., 2016). The analysis proves that GWAS of Arabidopsis accessions is a feasible approach to identify genetic loci that control the phenotypic variation in plant–microbe interactions. The challenge is to step beyond the relatively simple traits analyzed so far and to design screens that would allow to dissect the genetic architecture of the complex signaling and metabolic networks leading to variation in composition of root associated microbiota in different plant genotypes (**Figure 2**).

## Plant Root Exudates—A Source of Molecular Signals

### Metabolic Signals to Recruit Favorable Microbes

The growth of soil microbes is usually carbon-limited, so the high amounts of sugars, amino acids, and organic acids that plants deposit into the rhizosphere represent a valuable nutrition source (Bais et al., 2006). However, deposition of this labile carbon does not necessarily foster the recruitment of favorable microbes, because pathogenic strains can also use these molecules as growth substrates. Therefore, it can be postulated that plants have evolved recognition mechanisms to discriminate beneficial microorganisms from those that need to be repelled. In such a case, the specific molecules present in root exudates that contribute to shaping the microbial community structure are potential targets for plant breeding strategies that seek to engineer the rhizosphere microbiome. It has been shown that plant root exudates contain components used in belowground chemical communication strategies, such as flavonoids, strigolactones, or terpenoids (Bais et al., 2006; Venturi and Fuqua, 2013; Massalha et al., 2017). Studies on the microbiome of different plant species and accessions revealed strong variations, leading to the hypothesis that exudates are crucial in shaping plant–microbe interactions (Hartmann et al., 2009). Furthermore, it has been shown that plants specifically attract beneficial interaction partners via root derived signals (Neal et al., 2012; Lareen et al., 2016).

Up to now, most information about signal perception and transduction in plant–microbe interactions comes from the field of plant pathology, where plant receptor-like kinases (RLKs) play a major role (Antolín-Llovera et al., 2012). In case of mutualistic interactions, nodulation and mycorrhizal interactions serve as model systems to identify recognition mechanisms between plants and microbes (Delaux et al., 2015; Lagunas et al., 2015). In parallel to recognizing the microbial interaction partner by the plant, also microbes have to recognize their mutual interaction partner (the plant root). It is widely accepted that root exudates contribute to the establishment of the root microbiome (Massalha et al., 2017). The term “root exudates” describes the molecules that are selectively secreted by roots and distinguishes it from the sloughing-off of root border cells (Walker et al., 2003). The overall release of fixed carbon compounds (border cells and exudates) into the surrounding soil is termed as rhizodeposition (Jones et al., 2004; Dennis et al., 2010). Data about the amount of rhizodeposition range between 5 and 30% of the total amount of fixed carbon (Bekku et al., 1997; Hütsch et al., 2002; Dennis et al., 2010), which generally means a large loss of reduced-C for biomass and represents a considerable impact on the carbon budget of individual plants and also entire ecosystems (Badri and Vivanco, 2009; Bardgett et al., 2014). In a ^14^C approach, Hütsch et al. (2002) found remarkable differences in the amount of C-release among six different plant species ranging from 11.6 (wheat) to 27.7 (oil radish) mg C/g root dry matter. Also, the composition of these exudates varied between species, with oil radish exudates being rich in organic acids whereas pea exudates are rich in sugars. These data indicate that various plant species differentially modulate the chemical composition of their rhizospheres, which in turn might impact the associated microbial community. The recruitment of beneficial microbes might be crucial under environmental stress conditions such as nutrient limitation, pathogen attack, pests, high salt, or heavy metal stress.

### Issues to Consider When Analyzing Root Exudates

**FIGURE 3:**
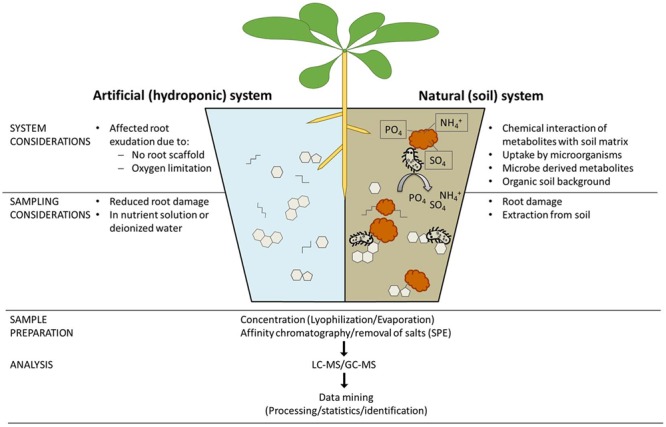
Technical considerations for analyses of root exudates. Comprehensive analyses of root exudate composition are crucial to advance our knowledge of plant–microbe interactions, but experimenters must consider the technical challenges at each step of the analytical workflow. First the growth system must be carefully considered, particularly whether to use soil or hydroponics, which each have advantages and disadvantages. At sampling, those using hydroponic systems must decide whether to collect the exudates in nutrient solution or deionized water, whereas those using soil systems must consider how to separate exudates from the soil matrix. During sample preparation, there are a multitude of options for sample concentration and clean-up, which will influence sample composition. Clearly, the mass spectrometry methodology used for data acquisition will play a crucial role in the workflow, because different setups allow the detection of different molecules. Finally, there is a growing awareness that data processing and analysis strategies also play a key role in shaping the derived data.

To fully understand the dynamic interactions between soil microbes and plant roots, it is necessary to elucidate the specific molecules within root exudates that can recruit favorable microbial strains. This is a challenging problem in analytical biochemistry, because various biological and methodological issues must be addressed to undertake biologically insightful analyses of plant root exudates (Rovira, 1969). Regarding cultivation, artificial plant growth systems cannot mirror the natural conditions in soil, but on the other hand, it is difficult to unravel the relevant communication signals occurring in soil, due to chemical interaction of metabolites with the soil matrix, and background metabolites released from decomposing organic matter or microbial exudation. Most analyses therefore settle on hydroponic cultivation, sometimes with an inert material to scaffold the roots. When sampling, the experimenter must choose whether to collect exudates in simple deionized water, or a more realistic medium containing mineral salts. Furthermore, it is effectively impossible to design an experimental approach that can differentiate exudates from sloughed-off border cells. A comprehensive summary on exudate collection and influences (e.g., pH, re-uptake by roots, incubation period) is presented in Vranova et al. (2013). For data acquisition, researchers are increasingly using unbiased mass spectrometry (MS) approaches such as gas chromatography (GC)-MS and liquid chromatography (LC)-MS. However, detection of all metabolites in a sample is impossible due to physiochemical biases imposed by the selected extraction method, sample clean-up procedure, matrix effects and analytical technique (Weston et al., 2015). Therefore, different methods have to be combined for a comprehensive view on the metabolite profile. The subsequent analysis of the derived MS data is a huge challenge, beginning with data processing algorithms that enable feature detection, peak alignment and different normalization methods. These normalization and scaling algorithms have a large impact on the outcome of an analysis (Worley and Powers, 2013). To validate the identity of specific mass spectral features, fragmentation data (MS^2^ or MS^n^) are acquired and compared against publicly available databases (Afendi et al., 2013; Misra and van der Hooft, 2016), or authentic standards (if available). Taken together, these challenges mean that comprehensive analysis of root exudates is not trivial (**Figure 3**).

### Recent Approaches to Analyze Root Exudate Composition

Various studies have described analyses of plant root exudates, with Phillips et al. (2008) developing a method to collect exudates from mature trees in the field, although microbial metabolism probably makes a significant impact upon this non-sterile system. That said, microbial nutrient uptake is an interesting aspect of plant–microbe nutritional interactions, with a fast degradation of flavonoid glucosides being observed by Carlsen et al. (2012) when comparing the flavonoid content in two soils and after different legume cultivations. To avoid microbial impact upon root exudate profiles, researchers have established diverse approaches of axenic hydroponic cultivation systems (Badri et al., 2008; Oburger et al., 2013; Strehmel et al., 2014), which are easier to control, even though they represent artificial plant cultivation systems and plant responses might also include stress reactions due to oxygen limitation and insufficient root support. Furthermore, hydroponics is well suited for sampling of exudates, as the total liquid can directly been taken for further sample preparation procedures and root damage is minimized. However, collection of exudates widely ranges in timescale and the used collection medium (nutrient solution or water). Badri et al. (2008) collected root exudates from *Arabidopsis thaliana* in nutrient solution for 3 and 7 days for analysis by LC-MS and revealed that most compounds are present only after the longer incubation period. It could be hypothesized that this observation is due to sloughed-off border cells. Nevertheless, they also compared the exudate composition against root composition and stated an 80% difference based on detected molecular masses (Badri et al., 2008). Also Strehmel et al. (2014) applied a 7-day collection period in nutrient solution to obtain sufficient amounts of exudates from *A. thaliana*. In contrast, Carvalhais et al. (2010) applied only a 6-h exudate collection period to *Zea mays* plants to minimize the effect of sloughed-off border cells, but used deionized water as collection medium. A similar approach has been used for barley root exudates that were collected for 4 h in deionized water (Tsednee et al., 2012). For a short-term exudate collection period from Arabidopsis a high amount of plants has been required to obtain sufficient amounts of exudates for LC-MS analysis (Schmid et al., 2014). A direct comparison of different plant cultivation and exudate collection techniques revealed a huge impact on metabolite patterns (Oburger et al., 2013). Especially, long incubations in deionized water may lead to overestimated exudation rates due to the high transmembrane gradient of solutes in low ionic strength medium (Neumann and Römheld, 1999; Oburger et al., 2013). To date, most published data on exudates concentrate on specific metabolite classes such as primary metabolites (Neumann and Römheld, 1999; Dakora and Phillips, 2002; Rudrappa et al., 2008; Carvalhais et al., 2010; Tan et al., 2013; Warren, 2015; Kawasaki et al., 2016), hormones (Foo et al., 2013), flavonoids (Graham, 1991; Hughes et al., 1999; Weisskopf et al., 2006; Cesco et al., 2010), or phytosiderophores (Oburger et al., 2014). Non-targeted metabolite profiling approaches of root exudates have been applied less frequently, although Strehmel et al. (2014) provided a comprehensive overview on secondary metabolites in Arabidopsis root exudates using LC-MS. In follow-up experiments, the data collection was complemented by GC-MS data and extended by a comparison of 19 Arabidopsis accessions (Monchgesang et al., 2016), co-cultivation with *Piriformospora indica* (Strehmel et al., 2016) and data from phosphate limitation (Ziegler et al., 2016). As MS technology continues to improve, it can be expected that more studies will undertake untargeted analyses of root exudate profiles.

### How Root Exudates Differ Across Plant Genotype and Nutrient Limitation

If root exudate profiles are to be a potential breeding target for increasing plant–microbe nutritional cooperation (Kuijken et al., 2015), then it must be first understood how exudate composition varies across genotypes or in response to nutrient deprivation. Recent studies have contributed to our knowledge of this phenomenon, with the effects of phosphate limitation investigated in Ziegler et al. (2016), and variation across accessions shown in Monchgesang et al. (2016) and also Micallef et al. (2009). Comparing exudate profiles across 19 natural Arabidopsis accessions showed a high natural variation for glycosylated and sulfated metabolites, such as flavonoids, glucosinolate degradation products, salicylic acid catabolites and polyamine derivatives (Monchgesang et al., 2016). Regarding root exudation changes induced by nutrient limitation, it seems that phosphate deficiency results in a higher abundance of oligolignols and a lower abundance of coumarins (Ziegler et al., 2016). Future experiments could investigate a different panel of plant genotypes, perhaps where previous experiments have defined a phenotypic difference that is potentially linked to root exudation profiles. One possibility involves comparing genotypes that have shown contrasting affinity to recruit favorable microbes (Haney et al., 2015), or genotypes that differ in their nutrient starvation responses (Ikram et al., 2012). Another option is root exudate profiling to analyze the phenotypic effects of mutants that were identified by GWAS studies (Wintermans et al., 2016).

### Specific Molecules in Root Exudates Linked to Plant–Microbe Nutritional Interactions

From our current knowledge of root exudates, is it possible to pinpoint a set of molecules that are particularly promising for recruiting favorable microbes to the rhizosphere? In legumes, it is well described that the flavonoid pathway has a huge impact on attracting rhizobia bacteria to roots and inducing *NOD* gene expression (Eckardt, 2006; Maj et al., 2010; Abdel-Lateif et al., 2012; Weston and Mathesius, 2013). Flavonoids are also crucial for hyphal branching and thus promoting mycorrhizal interaction (Abdel-Lateif et al., 2012; Hassan and Mathesius, 2012). Both of these interactions result in increased plant nutrient uptake, with mycorrhiza and root nodules boosting phosphorus and nitrogen, respectively. Perhaps other plants and soil bacteria have also tapped into this signaling pathway, and further analysis of root exudates will give us clues as to whether flavonoids play a role in this communication leading to increased nutrient uptake. From analyzing plant mutants, candidate genes that have been investigated so far are related to the transfer of metabolites into the rhizosphere (Badri et al., 2008), hormonal signaling (Foo et al., 2013; Carvalhais et al., 2015), or to biosynthesis, e.g., genes from phenylpropanoid pathway (Wasson et al., 2006; Zhang et al., 2009). These results give us some hints about candidate molecules that are exuded by plant roots to recruit beneficial bacteria, such as strigolactones and flavonols, and presumably further analyses will expand this list. To fully exploit this knowledge, then it is desirable to identify which microbial strains are recruited by these molecules, and what benefits they confer to the plant.

## Using Sequence Data to Predict Microbial Effects on Plant N, P, and S Nutrition

### Mechanisms of Microbial Nutrient Provision to Plants

As described above, plants give large amount of carbon away to the rhizosphere that nourishes soil microorganisms. So what do plants get back? In natural soils the vast majority of N, P, and S atoms are organically bound, while in the atmosphere the vast majority of N is contained in the N_2_ molecule. Due to the different metabolic capacities of plants and microbes, these nutrient sources are minimally bioavailable to plants, but can be metabolized by various soil microbes. This means that nitrogen fixing and nutrient mineralization processes carried out by soil microbes are crucial for plant nutrition in natural ecosystems, because these reactions metabolize recalcitrant forms of N, P, and S to liberate these elements for plant nutrition (Rovira, 1965; van der Heijden et al., 2008). It must be briefly stated that this established paradigm has been somewhat questioned in recent years, as several studies have demonstrated direct plant uptake of various organic-N forms (Nasholm et al., 1998; Paungfoo-Lonhienne et al., 2008). However, it is still generally accepted that microbes are better competitors for these nutrients due to the low diffusivity of organic-N molecules in soil, and the results of isotope labeling studies generally support the concept that most organic-N is first assimilated by microbial taxa, then subsequently assimilated by plants upon microbial turnover (Richardson et al., 2009; Kuzyakov and Xu, 2013).

Over several decades, the soil microbiology literature has accumulated a list of microbial metabolic processes that are linked to plant N, P, and S nutrition (summarized in **Table 1**). Commercially, the symbiotic association between legumes and bacteria is routinely exploited when field crops are inoculated with nitrogen-fixing rhizobia strains (Bashan et al., 2014). But, how can this phenomenon be refined and optimized for widespread use in more sustainable agricultural systems—not only for nitrogen fixation in legumes, but also for N, P, and S nutrition in non-leguminous crops? Given that different bacterial strains exhibit differing metabolic capabilities (Brader et al., 2014; Timm et al., 2015), coupled with the huge amount of genomic sequence data from soil microbes that has recently been generated (Bai et al., 2015; Muller et al., 2016), one possibility is to pinpoint the genes that encode metabolic pathways for agriculturally beneficial N, P, and S metabolism, and to boost the microbes that contain these specific genes in agricultural soils. This section will briefly outline what are the key known bacterial genes for boosting plant N, P, and S nutrition, and what strategies exist for promoting the abundance of these genes in agricultural soils.

**Table 1:** Key microbial metabolic processes related to plant nutrition.

Element	Biochemical	Microbial	Soil enzymology	Culture-independent	Culture-dependent
	process	genes	literature	literature	literature
Nitrogen	Nitrogen fixation	nifD, nifH, nifK		Reganold et al., 2010; Xue et al., 2013	Bremer et al., 1990
	Protein depolymerization	apr, npr, sub	Mader et al., 2002	Rasche et al., 2014	Kohler et al., 2007
	Urea catabolism	ureA, ureB, ureC	Dick et al., 1988; Bowles et al., 2014	Reganold et al., 2010; Fierer et al., 2012, Xue et al., 2013	Kohler et al., 2007
Phosphorous	Phosphate ester cleavage	phoA, phoD, phoX, ACPase, glpQ, ushA, appA, phyA, phyB	Mader et al., 2002; Garcia-Ruiz et al., 2008	Fraser et al., 2015	Kohler et al., 2007
	Phosphonate breakdown	phnJ, phnX		Bergkemper et al., 2016	
Sulfur	Sulfate ester cleavage	aslA, asfA	Garcia-Ruiz et al., 2008	Schmalenberger et al., 2008	Schmalenberger et al., 2008
	Sulfonate breakdown	ssuD			Kertesz and Mirleau, 2004

*Soil microbial metabolism boosts plant nutrition by converting recalcitrant forms of N, P, and S to forms that are more bioavailable for plant uptake. This table collates a set of well-known microbial metabolic processes that contribute to plant N, P, and S nutrition, highlighting the specific genes involved, and referencing specific studies that have conclusively shown this link.*

### Cultivation-Independent or Cultivation-Dependent Approaches

The major difficulty in investigating soil microbial communities is that only a small fraction of the inhabiting taxa can be cultivated in the laboratory, where experimenters can undertake detailed and controlled analyses (Pham and Kim, 2012). Therefore, the literature investigating root-associated microbes can be roughly divided into either cultivation-independent or cultivation-dependent studies. Generally, most cultivation-independent approaches extract root-associated microbes *in situ*, and then analyze the properties of this community. In contrast, cultivation-dependent approaches generally inoculate soil or root-associated microbes onto laboratory growth medium, before analyzing distinct strains that have been cultivated in the laboratory. For cultivation-independent approaches, techniques typically used to analyze soil microbiota often include: (1) 16S sequencing and PLFA measurements to infer taxonomic breakdown; (2) metagenomic, metatranscriptomic, or metaproteomic analyses to infer functional capacity of microbial communities; and (3) enzyme assays, respiratory measurements, or substrate utilization assays to measure functional activity of microbial communities. In cultivation-dependent approaches, the analytical possibilities are multitude—once root-associated organisms have been cultivated in a laboratory setting, they can be analyzed with any available technique. In the literature, there are many studies that use both cultivation-dependent and cultivation-independent techniques to draw links between microbial properties and plant nutrition (Kertesz and Mirleau, 2004; Richardson et al., 2009). But, can these acquired data inform the rational selection of microbes that will improve plant N, P, and S nutrition?

### Microbial Taxa and Metabolic Pathways in Cultivation-Independent Literature Linked to Plant–Microbe Nutrient Transfers

Several studies have used cultivation-independent approaches to investigate microbial community structure/function across soils exposed to different fertilization regimes (Allison et al., 2007; Williams and Hedlund, 2013; Bowles et al., 2014). Typically, these studies compare soil microbiota from highly fertilized soils against those from unfertilized or poorly fertilized soils, in situations where the low-fertilization regimes have encouraged mutualistic nutrient transfers between plants and microbes. From analyses of microbial taxonomy, it has been shown that the abundance of certain bacterial taxa is related to amount of applied fertilizer, with the copiotrophic phylum Actinobacteria being positively correlated with N fertilization, whereas the oligotrophic phylum Acidobacteria is negatively correlated (Ramirez et al., 2010, 2012). However, the results of a meta-analysis suggest that it is difficult to generalize a consistent response of microbial taxon abundance to N fertilization, because local environment and management play a dominant role in shaping microbial community structure (Geisseler and Scow, 2014). The work of Hartmann et al. (2015) examines bacterial 16S and fungal ITS2 sequences in a long-term field experiment comparing organic with conventional farming systems, showing correlations between taxon abundance and fertilization regime, with the bacterial Firmicutes phylum and several fungal taxa being more abundant on soils fertilized with manure.

The last 15 years has seen an exPLOSion in the number of rhizospheric microbiome sequencing studies, offering new taxonomical insights into the microbial communities associated with plants (Bulgarelli et al., 2013). However, the utility of taxonomic analyses for predicting microbial community function can be questioned, because in the bacterial literature it is becoming increasingly apparent that taxonomic groupings derived from 16S homology are imperfect predictors of a bacterial strain’s functionality (Beiko, 2015). Recent studies have sequenced the whole genomes of several closely related strains, and have discovered that although these strains are categorized as closely related due to the presence of a homologous core genome, in fact there can be considerable divergence in the accessory genome, meaning that the encoded functional capacities will also be significantly different. By sequencing the bacterial 16S gene, researchers infer the phylogeny of a strain’s core genome, but it can be argued that this gives little information about metabolic traits, because many of the key genes involved in N, P, and S metabolism are accessory genes, which are not taxonomically conserved due to the high prevalence of horizontal gene transfer between bacteria (Kumar et al., 2015; Young, 2016). Therefore, metagenomics studies that profile the abundance of all gene sequences (not just 16S) should have more power to unravel links between microbial genetics and plant nutrition, although it should be remembered that only a small proportion of the soil DNA pool is actively expressed. From metagenomic studies that compare the effects of different fertilizer inputs, it seems apparent that certain genes are more abundant in soils with lower fertilizer inputs, such as urea metabolism (Fierer et al., 2012) and unclassified metabolic genes (Leff et al., 2015). These genes are thus positioned as potential targets for improving the microbial provision of plant-bioavailable N, P, and S. However, the sheer complexity of the soil microbiome makes it difficult to draw mechanistic links between specific genes and ecosystem processes, which is one of the reasons why many researchers are adopting SynCom experiments that attempt to re-construct a simplified rhizosphere microbiome in a controlled setting (Busby et al., 2017).

In the soil biology literature, enzyme assays have established a set of enzymes linked to high-functioning soil microbiota, such as protease, urease, various phosphatases, and sulfatase (Garcia-Ruiz et al., 2008; Bowles et al., 2014). Therefore, the bacterial strains that possess the genes encoding these proteins are candidates for boosting nutrient transfers to plants. However, one challenge involves managing the stoichiometric availability of different nutrients to promote the activity of these enzymes. In the priming literature, it is generally accepted that soil microbiota are usually limited by the amount of labile C. Supply of labile carbon (e.g., root exudates) can relieve this limitation, such that N, P, or S then becomes the limiting nutrient, and microbes then express enzymes that can depolymerize recalcitrant forms of these nutrients. So, even if the soil microbiota contains strains with genes encoding the aforementioned enzymes linked to soil health, soil conditions must be optimized for these microbial proteins to be expressed and active (Paterson, 2003). Another method to measure metabolic capacity of soils involves community level physiological profiling assays, which measures substrate degradation affinity across different fertilization regimes, although usually these assays are designed with an emphasis on degradation of C-sources rather than sources of N, P, and S. This technique has been applied to soil receiving different fertilization practices, and it has been shown that the capacity to degrade a diverse range of substrates is correlated to other aspects of soil health, such as organic carbon content and disease suppression (Perez-Piqueres et al., 2006; Nair and Ngouajio, 2012; Dumontet et al., 2017). Perhaps future studies could modify this approach to develop assays that measure the capacity of soil microbes to degrade various sources of N, P, and S. However, the mechanistic insight derived from these assays is sometimes questioned, because they measure the capacity of soil microbiota to grow on specific nutrients under laboratory conditions. This setup may therefore select for a small number of fast-growing taxa and not correlate with the *in situ* activity of these substrate degradation pathways (Ros et al., 2008; Rutgers et al., 2016).

### Microbial Genes in Cultivation-Dependent Literature Linked to Soil Fertility

It can be posited that soils with high rates of microbial N, P, and S cycling should harbor microbes with specialized genes encoding these traits, and therefore that microbial strains isolated from these soils should possess useful metabolic attributes for boosting plant nutrition. Interestingly, *Rhizobia* strains isolated from N-fertilized soils exhibited a lower capacity to promote plant growth compared to strains isolated from adjacent unfertilized plots (Weese et al., 2015), indicating that the management history of the isolation site impacts the degree of mutualism in the resulting isolate. In the P literature, a similar phenomenon has been observed, with phosphate mineralization being more common in isolates from soils where bioavailable-P was less abundant (Hu et al., 2009; Mander et al., 2012). For sulfur, one example is from soils from the Rothamsted Broadbalk experiment, where different S-fertilization practices had led to fields that exhibit high versus low sulfatase activity. Bacterial strains were isolated from these contrasting soils, and functional assays such as enzyme measurements and growth on minimal media revealed that the strains isolated from low-SO_4_^2-^ soils contained several mechanisms for depolymerizing organic-S (Schmalenberger et al., 2008). Together, these results imply that research programs seeking “elite” microbial strains that can maximally boost plant nutrition could begin with inocula from sites that favor plant–soil feedbacks, such as unfertilized soils or organic farms (Xia et al., 2015; Melo et al., 2016).

### Microbial Strains That Promote Plant Growth by Enhancing N, P, and S Nutrition

To truly be useful in an agricultural setting, it must be proven that candidate growth-promoting strains can be re-inoculated onto plants, successfully colonize the rhizospheric niche, and then mediate nutrient mobilization that benefits plant growth. This can be tested through plant–microbe interaction assays, where candidate strains are tested for their ability to promote plant growth and nutrient acquisition (Ahemad and Kibret, 2014). Once again, this research field is most mature for the case of nitrogen-fixing *Rhizobia*, where decades of research have endeavored to define the optimal inoculation practice, searching for the right combination of plant genotypes and rhizobia strains to suit specific climates and soils (Lindstrom et al., 2010). Regarding the taxonomy of nitrogen-fixing symbioses, it should be mentioned that nitrogenase genes are present in diverse bacterial taxa (Gyaneshwar et al., 2011), and that non-leguminous plants have been documented to host N_2_-fixing bacterial strains (Santi et al., 2013), perhaps implying that other plant–microbe combinations (not just legumes and *Rhizobia*) could be similarly optimized to promote nitrogen fixation (Mus et al., 2016). There are also reports of plant growth promotion via microbial mobilization of other nitrogen sources, shown by higher yield in plants inoculated with bacterial strains (Shaharoona et al., 2008; Adesemoye et al., 2009), although for one of these experimental setups, it seems that the source of this N was directly from the ammonium sulfate fertilizer rather than from organically bound soil N (Adesemoye et al., 2010). It has been shown that unsterilized grass seeds can better access protein-N compared to sterilized seeds, but the specific strains that provide this service were not elucidated (White et al., 2015). The ability of the fungi *Glomus intraradices* to transfer organic nitrogen to plants has also been shown (Thirkell et al., 2016), suggesting that future experiments could focus on documenting other fungal strains with this capacity and characterizing the relevant genes and mechanisms. For phosphorous, the literature contains a large number of reports of both fungal and bacterial strains with the capacity to solubilize inorganic P, and also many reports of strains that can mineralize organic P (Plassard et al., 2011; Ahemad and Kibret, 2014). Many of these P-mobilizing strains are also characterized as growth promoting microbes, but microbial promotion of plant growth can operate through a wide variety of mechanisms, and sometimes it is not conclusive that P-mobilization is responsible for the plant growth promotion elicited by these strains (Richardson and Simpson, 2011). For sulfur, studies of a plant growth-promoting *Pseudomonas* strain have used genetic knockout of the sulfonate monooxygenase enzyme to show that organic-S mineralization accounts for some fraction of the growth-promoting phenotype (Kertesz and Mirleau, 2004).

The research field is beginning to build large collections of genomically sequenced bacterial isolates that can be re-assembled into SynComs (Bai et al., 2015). Xia et al. (2015) isolated endophytic bacterial strains from plants grown under organic management, and showed that over half of these strains can boost tomato growth in a greenhouse experiment. This high proportion of growth-promoting isolates shows that the capacity to promote plant growth is widespread amongst plant-associated bacteria, but to gain a mechanistic understanding into how these growth-promoting effects are manifested, it will be necessary to conduct detailed investigations into the genetics, biochemistry and physiology of these growth-promoting strains. Furthermore, microbial community experiments should also consider how the interaction between different strains affects plant growth promotion. Such knowledge will enable the rational selection of growth-promoting strains and communities, driven by defined genetic and biochemical mechanisms.

## A Growing Community: Challenges and Perspectives in Modeling Plant–Microbe Interactions

With the vast quantity of data being generated by high-throughput experimental techniques, new opportunities are arising to integrate theoretical and computational approaches with experiments. Potential synergies include designing hypothesis-driven experiments based on the results of modeled scenarios, or using modeling as a tool to mechanistically interpret the results of high-throughput experiments (O’Brien et al., 2015). In the bacterial field, there is currently a major initiative to integrate genomic sequencing data with computational modeling, in order to predict the function of individual bacterial strains and whole bacterial communities (Blaser et al., 2016). Already, microbial engineering is a mature technology that exploits the power of computational modeling to optimize strains and communities for industrial processes like bioremediation and fermentation (Perez-Garcia et al., 2016). Regarding the study of the rhizosphere microbiome, over the past 15 years this field has accumulated a huge volume of sequencing data, but these data are generally descriptive, and to date they have not been used to significantly further our mechanistic understanding of nutrient exchange processes in the rhizosphere. We see mathematical modeling as the most promising approach to bridge this gap, and in the following section we want to elucidate through examples the impact mathematical modeling can have for future improvement of rhizosphere interactions to boost plant growth.

One of the principal aims of mathematical models is the reduction of complexity in order to capture the fundamental principles behind the phenomena of interest. There is nowadays a positive trend in biology to develop predictive models of the system under study, with some disciplines at a more advanced stage than others in the integration of theory and experiments. The study of microbial communities is a clear illustrative example of a field where computational approaches are essential (Widder et al., 2016). While bioinformatics is instrumental to analyze high throughput data from meta-omics experiments, theoretical models are developed to gain mechanistic understanding of complex biological systems. Mathematical descriptions of population dynamics were pioneered in the 19th century by Verhulst’s law of logistic growth (Verhulst, 1838) and are now a fundamental part of ecology (Murray, 2002). In the same way, over the last five decades models of metabolism have started converging into sound mathematical methods to investigate cellular functions (Heinrich and Schuster, 1996). It is also relevant to point out that another characteristic of mathematical models is that, as long as the described mechanism holds true, they can be easily generalized to different organisms. Therefore, as an example, a model originally built to describe bacteria might be also applied to other microbes.

Theoretical biology is a rapidly expanding field and it is nearly impossible to condense and classify it in few words, but we can roughly point out three main classes of methods: kinetic models, stochastic models, and network-based models. It is important to point out that depending on the system and phenomena under study, certain modeling techniques will be more suited than others. Kinetic models are dynamic and deterministic, typically constructed as systems of differential equations (Holmes et al., 1994), solved nowadays with computational integration algorithms. These models can offer precise predictions but require either *a priori* knowledge or inference (e.g., through a fit to data) of the equation parameters. Typically, these models are well suited to describe small-scale metabolic pathways where most enzyme kinetic constants are measured and few parameters are reasonably constrained and then fit. Stochastic models encompass any representation that implements some random components with a Monte Carlo procedure and they are needed to capture effects like noise or individual variability. A widely used method is the Gillespie algorithm (Gillespie, 1977) which simulates in randomized steps the evolution in time of, e.g., a chain of biochemical reactions, each of which has an associated probability to happen. Under the general term network-based models (Milo et al., 2002; Feist et al., 2009) we include different static methods that have in common the treatment of metabolic pathways as networks, where metabolites are connected to reactions either as substrates or products. Reactions can be represented either as *edges* connecting *nodes* (the metabolites) or, in bipartite networks, as a disjoint set of nodes (the enzymes) that connect to the metabolites nodes via edges carrying, e.g., stoichiometric information. Reversibility of a reaction is determined by physical principles and is taken into account in directed networks.

Mostly, systems of plant–microbes have been considered in light of host–pathogen interactions. The classic zig-zag model (Jones and Dangl, 2006) is an illustrative scheme proposed to explain the function of the plant immune system in response to pathogens. It distinguishes two branches of the plant immune system, one reacting to microbe-associated molecular patterns with pattern-triggered immunity (PTI), the other responding to effectors trying to suppress PTI with effector-triggered immunity. This picture is, however, a purely expository model and is not suited to capture the interaction dynamics we are interested in (Pritchard and Birch, 2014). Indeed, if the objective is to build a predictive model of plant–microbe interactions, and in particular beneficial ones, the fundamental ingredients include: (i) the actual molecular factors driving the interactions, like the ones discussed in the previous sections of this review; (ii) environmental conditions inducing different reactions, e.g., to stresses; (iii) temporal and spatial scales of the phenomena under study. While it would be nice to have a single model drawn from a universal theory of plant–microbe interactions, we are mostly at the stage where mathematical models are first needed to answer a limited number of focused and well-defined biological questions. With the current advancement in “omics” experimental techniques and with the development of computational methods to understand metabolic pathways, quantitative models of plant–microbes ecosystems at the molecular level become an appealing possibility.

**FIGURE 4:**
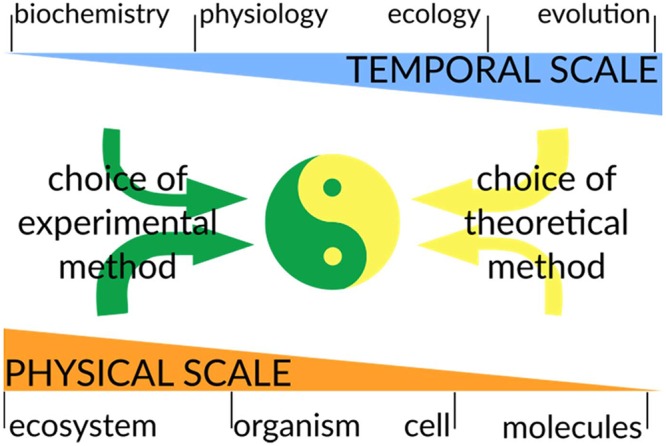
Considerations for combining modeling and experimental approaches. A major goal in biology is to integrate computational predictions with experimental data to generate predictive models of biological systems. While the temporal scale is an intrinsic property of the phenomena under study, the choice of the physical scale of the model/experiment is chosen by the scientist: one can investigate soil microbial communities at an ecosystem macroscale or at the DNA sequencing level. In general, the physical scale will have a strong impact on the choice of experimental technique, while the temporal scale will mostly influence the experimental design (this is represented by thicker/thinner arrows). On the contrary, theoretical methods will be highly constrained by the temporal scale to be modeled, while they are rather flexible regarding the physical scale to be operated at. In a truly interdisciplinary approach, the experimental and theoretical methods have to be planned together to ensure that the reciprocal results are compatible and can be integrated. This will allow further improvements in both experimental design and model development.

In the following section, we will outline some examples that illustrate how different techniques can better capture different phenomena. It is essential, when developing a theoretical model, to understand at which temporal and spatial scale the biological system operates. The temporal scale is an intrinsic property of each biophysical process and therefore sets specific limits on the mathematical method to use. On the other hand, the spatial scale to investigate is chosen by the modeler based on the biological question to address, since we can chose to describe the same microbial community as a single metabolic unit or as a population of individual organisms (**Figure 4**). These choices are critical since they will determine at the same time the degree of complexity of the model (an Earth-wide ecosystem model can be simpler than a model of a *Escherichia coli* metabolism) and the requirements for the integration of experimental data (Succurro et al., 2017).

### Dynamic Models of the Rhizosphere

The rhizosphere comprises the shared environment between the plant roots and microbes. Understanding the dynamics of nutrient competition in the rhizosphere is an important aspect to identify the driving mechanisms of community assembly. The work of Darrah (1991a,b) presents one of the first differential equation models of microbial population dynamics in the rhizosphere, parameterizing how root growth and exudation of soluble C fuels microbial growth at specific sites. These models accounted for root elongation and release of soluble organic compounds which diffuse through the surrounding area populated by bacteria. The simulations showed how different exudation patterns can control the distribution of bacterial biomass. Being based on simple equations representing the spatial gradient of soluble substrates, this model is easily expanded to include the presence in the rhizosphere of other solutes like minerals essential to plant growth (Darrah et al., 2006). Recently, a similar approach was used to model the colonization of the root tip by bacteria, introducing bacterial motility and under the assumption that carbon is the growth-limiting nutrient (Dupuy and Silk, 2016).

Borrowing concepts from microeconomics, Schott et al. (2016) modeled the nutrient trade system between plants and arbuscular mycorrhizal fungi as a minimal network, and investigated the partnership relation in terms of costs and revenues. First, they demonstrated that a simple model where the nutrient exchange via the periarbuscular space is only regulated by proton pumps (H+-ATPase) and nutrient transporters (H+/sugars and H+/phosphate cotransporters) could reproduce the situation where C and P exchange is stable at the thermodynamic equilibrium. This result shows that this small set of transporters can explain C and P fluxes between the plant and the fungi, although the presence of further transporters cannot be excluded from this result. Interestingly, while the observable highly cooperating behavior might suggest altruistic behavior in a “reward-based” system, the symbiosis is the result of a selfish optimization of personal benefit. A change in the costs to revenues ratio, for example, a well-fertilized plant that places a lower value on fungal-derived P would therefore lead to a different nutrient exchange rate (Schott et al., 2016).

Soil nutrient fluxes also play a role in more global scenarios. Earth system models are extended climate models that include the effects from biogeochemical cycles, and different competition theories can be embedded in them. Recently, Zhu et al. (2016) demonstrated that a kinetic model considering plant, microbes and nutrients as a coupled network, and based on the principle that nutrient uptake rate is controlled by specialized transporter enzymes, can reproduce data of N competition in a grassland ecosystem where other competition theories fail (Zhu et al., 2016).

### Genome-Scale Metabolic Network Models

Differential equation models are perfectly suited to simulate dynamic processes like enzymatic or diffusion reactions, but rely on fixed parameters that characterize specific states. In principle it would be possible to obtain exact kinetic models of metabolic networks at the “genome scale” (genome-scale models, GEMs) if we were to know the function and the kinetic parameters of each enzyme in the picture. Such models would be, however, computationally expensive to solve and difficult to manage in terms of number of equations, variables and parameters. Instead, a convenient approach is the reconstruction of GEMs from automated genome annotation and their representation as networks (Thiele and Palsson, 2010). In an automated process of mining the information from databases (Henry et al., 2010), the enzymes encoded in the genome are assigned metabolic functions. The resulting GEM, however, still requires subsequent careful manual curation as a consequence of possible inaccurate annotations besides our incomplete knowledge of genes associated to metabolic activities.

Once a metabolic network has been reconstructed, it can be translated into a stoichiometric matrix. This mathematical formalism, connecting reactions to metabolites *via* the stoichiometric coefficients, presents itself to a number of methods developed to study the function of biochemical pathways (Fell and Small, 1986; Varma et al., 1993; Papin et al., 2004). Constraint-based models investigate the distribution of reaction fluxes along the directed metabolic network (first constraint, imposing irreversibility or reversibility under physiological conditions from thermodynamics principles) under the assumption that the system is at a steady state (second constraint, meaning that there is internal balance of production and consumption of metabolites) and that enzymes operate at limited capacities (third constraint, setting upper or lower bounds to the rate of an enzymatic reaction based on experimental observations). This set of constraints leads to a narrower space of possible solutions for the metabolic fluxes, but a further step is needed in order to identify a unique solution. A widely used method is flux balance analysis (FBA) (Orth et al., 2010), where an optimization problem is defined by introducing an objective function (usually a linear combination of metabolic rates) either to be maximized (like in the case of biomass as objective function) or minimized (like in the case of total sum of cellular fluxes).

GEMs are in general compartmentalized according to the cellular structure. The simplest division, typically for prokaryotic organisms, is between an external and an internal (cytosolic) compartment, while plant GEMs will have compartments for different tissues as well. Exchange of metabolites between compartments happens through transport reactions, which are not always correctly annotated and often added to the network during the gap-filling process of the reconstruction. An exchange flux with the external compartments is equivalent to an uptake or secretion rate.

The GEM of the nitrogen fixing bacterium *Rhizobium etli*, a known symbiont of legumes, was studied with constraint-based modeling to understand its physiological capabilities during the third developmental stage of the symbiosis in the plant nodule, when it differentiate into a bacteroid and provides N to the plant (Resendis-Antonio et al., 2007). The authors explored how N fixation can be influenced by succinate (the carbon source provided by the plant to the bacteroid) and oxygen (nodules protect the nitrogenase enzyme from oxidative damage by providing a microaerobic environment). For a given oxygen uptake rate, an increase in succinate uptake rate leads to higher symbiotic N fixation, until a threshold value in succinate uptake rate is reached. After that an inhibitory effect, caused by insufficient oxygen for succinate reduction, drastically lowers N fixation rate. These kinds of studies, quick to perform *in silico*, can be easily transferred to other pathways of interest to generate hypothesis relating environment to metabolism.

Available GEMs can be browsed at the BioModels database (Le Novere et al., 2006), where most of the reconstructed models are deposited and usually classified according to their level of curation. We have to point out that efforts are still on-going to converge on model standards, and the trustworthiness of simulation results depends on the quality of the GEM (Ravikrishnan and Raman, 2015; King et al., 2016).

### From Single Species to Soil Communities

Even without considering the associated plant, mathematical modeling of microbial consortia is a very diverse research field where multiple methods can (and should) be applied. For a wide overview we point the interested reader to specific reviews (Song et al., 2015; Zomorrodi and Segre, 2016), while here we will focus on metabolic models, pointing out advantages and limitations. A clear asset of methods like FBA is the possibility of manipulating GEMs with ease, obtaining direct information on active reactions. There are straightforward observable quantities that can be experimentally measured to inform or to challenge the model prediction, like nutrient uptake, oxygen levels, or biomass growth. However, as mentioned before, the simulation results are determined by the choice of an optimality principle, which is still an extremely subjective and approximate choice (Schuetz et al., 2007). If the concept of optimality is debatable for a single organism undergoing metabolic regulation by circadian rhythms and environmental factors, is it easy to imagine that defining optimality in a microbial community, where many other dynamics enter the picture, is far from trivial.

Considering the temporal scale of constraint-based models, the steady state condition implies that we obtain a static representation of the mass-balanced metabolic system. If we want to recover some dynamic information, we can consider that metabolism adjusts quickly to small perturbations and that it will be at a “quasi-steady-state” compared to slower external processes (Harcombe et al., 2014). Moving to the spatial scale, there is no unique way to build a community metabolic network model (Perez-Garcia et al., 2016). Generally speaking, we can consider the results on a single organism metabolic model as a proxy of a colony of a single species, and different approaches have been proposed to describe communities of various species. For example, one possibility is to lump the metabolic pathways of each species together, obtaining a single “super-organism” where the biochemical reactions are acting together to optimize a common objective function. Another approach is to build a compartmentalized model, where each species is a separate compartment and transport fluxes allow the exchange of metabolites between them, and the modeler can choose to implement a common objective function or differentiate it among the organisms. While both choices are valid, one strategy can be more appropriate than the other in the specific context drawn by the biological question under investigation.

The recent work of Pfau (2013) is, to our knowledge, the first example of a metabolic network model of a plant–microbe system (Pfau, 2013). The authors first reconstructed the GEM of *Medicago truncatula*, an annual legume used as model plant for studies on legume–rhizobia symbiosis, obtaining a highly curated multi-tissue model describing root and shoot. The metabolic network model of the nitrogen fixing symbiont *Sinorhizobium meliloti* was then connected to the root tissue model through exchange reactions extracted from literature. Simulations on the GEM of plant and symbiont under different N availability quantified the benefit, in terms of growth, for the symbiotic partners. The insight on exchange fluxes showed that *S. meliloti* exported only alanine as N source to the plant. A simulation repeated for an alanine dehydrogenase knock-out version of the bacterium showed that N was provided to the plant as ammonia, with an increased need for oxygen. These first results, backed-up by experimental data, already show the potential of theoretical models to provide testable hypotheses and mechanistic understanding of symbiotic interactions involving nutrient exchange.

## Conclusion

**FIGURE 5:**
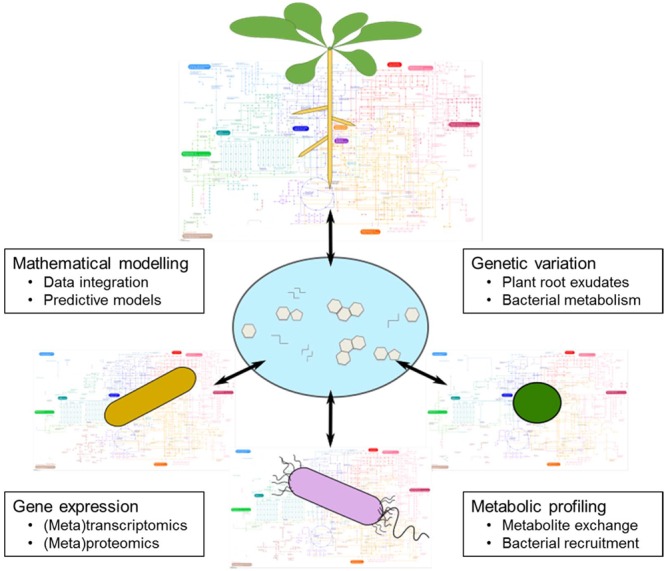
Metabolic interconnections between plants and soil microbes. A major research goal is to generate a mechanistic understanding of the metabolic interactions between plants and rhizosphere microbes. This scheme illustrates a set of research themes that will deepen our knowledge of this phenomenon. Regarding genetic variation, a major goal is to define the genetic elements responsible for variation in plant root exudate composition, and also the genes that encode for desired bacterial metabolic pathways. Metabolic profiling is a key tool in this field, because it can provide direct measurements of root exudate composition and be used to identify specific metabolites linked to bacterial recruitment. Gene expression studies of plant–microbe interactions should reveal the key genes that are induced or suppressed during favorable nutritional interactions. Finally, mathematical modeling is a crucial tool to integrate the derived data, with the goal of building predictive models that enable us to rationally design agricultural systems for beneficial nutritional interactions between plants and microbes.

For plants to access recalcitrant soil-borne nutrients, they are dependent upon the metabolic activities of soil microbiota. Considering the environmental damage associated with current fertilization practices, a current research priority is to optimize plant–microbe nutritional interactions for more sustainable agricultural systems. However, the specific mechanisms governing the assembly of the plant microbiome and its modulation according to plant nutritional status are extremely complex and difficult to predict. Despite the experimental challenges described here, we argue that many important jigsaw pieces have been identified that will enable us to understand the mechanisms governing dynamic plant–microbe interactions (**Figure 5**). We know that although soil is the major determinant of the microbial community associated with plant roots, plants have a significant effect on taxonomic assembly. Therefore, comparative genetic approaches such as GWAS promise to identify plant genes and processes important for controlling how plants shape the rhizospheric microbiome. Particularly interesting are genes in plant metabolic pathways that affect the composition of root exudates and thus the actual signals in the rhizosphere. Therefore, continued progress in our capability to collect and analyze exudates will be important for assessing the molecules plants use for communication with soil microbes, and also the pathways the microbes use to decrypt these signals.

Recent genomic studies are beginning to reveal the specific microbial strains that contain metabolic pathways favorable for plant nutrition (Muller et al., 2016). The big question, however, remains: to what extent can plants attract specific microbes for specific environmental/nutritional conditions? For a number of microbes the mechanisms of their plant growth promoting effects have been deciphered, however, it seems that almost every individual organism uses different processes. How do these various mechanisms integrate in real soil with a multitude of taxa? Rhizosphere microbiome research has already started to move from description of the communities to assessing their dynamics due to changes in environmental conditions (Busby et al., 2017). However, still very little is known about which specific microbial strains are the key contributors to plant nutrition, or about how nutrient availability affects the composition of the rhizospheric microbiome. How can we merge the progress in the individual areas of research to obtain an integrated view? Already we can design experiments with synthetic microbial communities to identify processes important for the establishment of effective communities using different plant genotypes (Castrillo et al., 2017). However, there is still an enormous knowledge gap regarding a sound theory of plant–microbe interactions. The huge volume of data obtained from the characterization of the microbiome is clearly calling for a modeling approach, e.g., to construct nutritional networks integrating metabolic pathways of plants and microbiota. Such models would allow researchers to assemble various SynComs with defined metabolic capacities, and facilitate dissection of the mechanisms that shape microbial community composition and function. This knowledge could mechanistically guide the highly promising approaches to use microbes for more sustainable plant nutrition.

## Author Contributions

RJ, MP, AS, AK, and SK together designed the scope of the manuscript, wrote individual chapters, and prepared the figures.

## Conflict of Interest Statement

The authors declare that the research was conducted in the absence of any commercial or financial relationships that could be construed as a potential conflict of interest.
